# N-3 Polyunsaturated Fatty Acids in Elderly with Mild Cognitive Impairment: A Systemic Review and Meta-Analysis

**DOI:** 10.3233/JAD-220863

**Published:** 2024-04-22

**Authors:** Lei Yang, Fengxue Zhao, Yadi Sun, Ziyi Wang, Qianwen Li, Hao Wang, Ying Lu

**Affiliations:** a School of Nursing, Xinxiang Medical University, Xinxiang, Henan, China; b Department of Nursing, Henan Provincial People’s Hospital, Zhengzhou, Henan, China; c Department of Nutrition, Henan Provincial People’s Hospital, Zhengzhou, Henan, China; d Department of Nutrition, Precision Nutrition Innovation Center, College of Public Health, Zhengzhou University, Zhengzhou, Henan, China

**Keywords:** Elderly, meta-analysis, mild cognitive impairment, n-3 polyunsaturated fatty acids

## Abstract

**Background:**

Mild cognitive impairment (MCI) is the prodromal stage of dementia. In this stage, reasonable intervention measures can help to delay the decline of cognitive function. Supplementation of n-3 polyunsaturated fatty acids (n-3PUFAs) may be beneficial to delay the decline of cognitive function in the elderly.

**Objective:**

To investigate the effectiveness of docosapentaenoic acid (DHA) or/and eicosapentaenoic acid (EPA) supplements in the elderly with MCI.

**Methods:**

Eight electronic databases, PubMed, Cochrane Library, Embase, VIP, SinoMed, Web of Science, CNKI, and WANFANG DATA, were searched for related articles from inception until January 2022. Subgroup analyses and sensitivity analyses were performed to detect confounding variables. Standardized mean differences (SMD) with 95% confidence intervals (CI) were determined. Heterogeneity was evaluated by I^2^ statistics. Publication bias was detected using funnel plots. Stata12.0 was used for Begg’s and Egger’s test to quantify whether publication bias. Linear relationship between global cognition and covariates was examined in meta-regression analysis.

**Results:**

Twelve studies (*n* = 1,124) were included. The methodological quality of research is mostly medium. Compared with placebo, n-3PUFAs supplements have benefits on global cognition [SMD = 0.51, 95% CI(0.12, 0.91), *p* = 0.01]. No significant differences were observed between intervention group and placebo on language fluency, executive functions, and depression.

**Conclusion:**

Our findings indicated DHA and/or EPA supplements have benefits on global cognition, and it may also reduce the level of blood amyloid-β (Aβ)-related biomarkers (e.g., Aβ_40_, Aβ_42_) and inflammatory factors (e.g., 1L-6, 1L-10). Since there are only two relative articles, more research is needed in the future to clarify the relationship.

## INTRODUCTION

As a major public health issue, the prevalence of dementia continues growing over time in the aging population [1]. The numbers of dementia are expected to reach 75 million by 2030, and 131 million by 2050, with the greatest increase expected in low-income and middle-income countries [2]. Alzheimer’s disease (AD), the most common type of dementia, is a heterogeneous disease with a complex pathobiology. Currently, AD still lack effective treatment, for numerous phase III clinical trials have failed to demonstrate benefits [3]. Therefore, early prevention and intervention are very important.

Mild cognitive impairment (MCI) is a transitional stage of normal aging and dementia. It is mainly manifested as early cognitive decline, but the daily living ability is basically normal and has not yet reached the diagnostic criteria for dementia [4]. Neuropsychiatric symptoms like depression, irritability, apathy, anxiety, agitation, and sleep problems are highly prevalent in MCI patients and associated with subsequent cognitive deterioration [5]. MCI affects 10–15% of the population over age 65. The failure of drug trials in AD treatment makes investigators try to delay MCI progressing into dementia, by which the prevalence and costs of dementia would be reduced profoundly [6]. A practice guideline indicated MCI prevalence was 6.7% for ages 60–64, 8.4% for 65–69, 10.1% for 70–74, 14.8% for 75–79, and 25.2% for 80–84. Cumulative dementia incidence was 14.9% in individuals with MCI older than age 65 followed for 2 years, and there is no effective pharmacologic treatments for MCI [7]. However, lifestyle modifications, including diet, exercise, and cognitive stimulation, may be effective to delay cognitive decline [8]. The other reversible factors of MCI include depressive symptoms, chronic diseases, and participation in social activities [9, 10].

For elderly, it is important to maintain health by nutritional supplement. Appropriate dietary measures or supplementation with specific micro- and macro-nutrients might provide novel ways to prevent or manage cognitive decline and dementia. For example, n-3 polyunsaturated fatty acids (n-3PUFAs) metabolism plays important roles in human health and disease [11]. The sources of fatty acid (FA) are various, including endogenous synthesis and exogenous uptake. Among them, n-3PUFAs docosahexaenoic acid (DHA) and eicosapentaenoic acid (EPA) can only be obtained from the diet and are called essential fatty acid [11, 12]. DHA is required throughout the life cycle for maintaining brain functions. DHA facilitates the development of neurons in brain, and cerebral DHA mainly come from the circulation. Therefore, circulating plasma DHA is significantly correlated with cognitive abilities during aging and is inversely associated with cognitive decline [13, 14]. DHA is essential to brain development, whereas EPA seems more influential on behavior and mood [15]. Meta-analyses suggested that high EPA supplements may be beneficial in managing depression symptoms. Moreover, DHA can also be synthesized from EPA, and therefore EPA level and EPA/DHA turnover are important for brain DHA [16].

Currently, several epidemiological evidence suggested that increased polyunsaturated fatty acids (PUFA) uptake may protect against cognitive decline [17, 18]. Nevertheless, the outcomes of trials with DHA or/and EPA supplementation on mild cognitive impairment are controversial. A meta-analyses from Alex et al. showed n-3PUFAs have no effect on global cognitive function, only memory function showed a mild benefit in non-demented adults [19]. Martí et al. reported that n-3PUFAs supplementation might have positive effects on preventing cognitive decline in aged adults [20]. Yet, Balachandar et al. provided current evidence that do not support the protective roles of DHA supplementation in age-related cognitive decline (including memory, executive function, attention, and working memory) [21]. There was only a meta-analysis about n-3PUFAs on MCI, which showed the beneficial effect in elderly with MCI [22]. Given that MCI patients are often accompanied with neuropsychiatric symptoms (such as depression), Liao et al. showed a beneficial effect of n-3PUFAs on depression symptoms [23]. Yet, it is unclear whether n-3PUFAs supplementation improves depressive mood in MCI patients and the other benefits of n-3PUFAs supplementation for MCI patients.

As mentioned above, although there are some related meta-analyses about n-3PUFAs on cognitive function currently, most of them did not focus on MCI. Moreover, the effects of n-3PUFAs on cognitive function are still controversial, due to the differences in subjects, age, and outcome indicators. Therefore, we conducted a comprehensive search to investigate the effect of n-3PUFAs supplements on elderly patients with MCI at cognitive function (global or individual domain score), mood and blood biomarkers levels by a systematic review and meta-analysis.

## METHODS

The review was conducted according to the “Preferred Reporting Items for Systematic Reviews and Meta-Analyses” (PRISMA-P) guidelines [24] and the review protocol was registered at PROSPERO (registration number: 2022 CRD42022340719).

### Search strategy

Related articles reported the effect of DHA alone or with EPA, as supplements sources on MCI elderly were searched from PubMed, Cochrane Library, Embase, VIP, SinoMed, Web of Science, CNKI, and WANFANG DATA in English and Chinese from inception until January 2022. The following search terms were used: “Cognitive Dysfunction OR Cognitive Impairment OR Mild Cognitive Impairment OR MCI” AND “Fatty Acids OR n-3 Fatty Acids OR omega-3 Polyunsaturated Fatty Acid OR DHA OR EPA” AND “randomized controlled trial OR randomized OR RCT”. Reference lists in the included studies were also searched and also referred recent reviews to find relevant studies.

### Selection criteria

Studies were eligible for inclusion if they met the following criteria: 1) Intervention measures and study design: randomized controlled trials published in Chinese and English on supplementation of n-3PUFAs including DHA and EPA alone or in combination as the main interventions, the control group treatment method can be no treatment or basic treatment; 2) MCI adults aged 60 years or older; 3) clear design for n-3PUFAs supplementation with EPA and DHA in combination or alone and time and dosage; 4) Diagnostic criteria: the MCI diagnostic criteria proposed by Peterson et al. [25] or diagnosed with MCI based on clinical diagnosis, but they were required to meet the most basic criteria: cognitive decline but did not meet the diagnostic criteria for dementia, activities of daily living were generally normal.

Studies were excluded if they 1) were conducted on patients with Alzheimer’s disease, dementia, or other neurological conditions such as Parkinson’s disease, epilepsy, stroke, head injury, substance abuse and so on; 2) non-randomized controlled trial, animal trails and other experimental design types; 3) articles without full texts.

### Data collection process

Two authors carefully and independently reviewed the full text of selected eligible studies. The extracted information of included studies consisted of first author’s name, country, diagnostic criteria, ample size, publication year, age, duration of intervention, supplementation doses of EPA and DHA conclusion, cognitive domains, mood, blood biomarkers, level of DHA/EPA, and adverse event. If there was any disagreement among two reviewers, the report was discussed with the third author and the three authors reached a consensus finally.

### Outcome measures

The outcome of this study including 1) global cognitive function before and after supplementation of n-3PUFAs compared with placebo; 2) individual cognitive domains like executive function, memory, language; 3) mood: depression; 4) proportion of DHA and EPA compared to the placebo groups and other related results.

### Risk-of-bias assessment

This study was evaluated according to the Cochrane literature quality assessment tool including “sequence generation,” “allocation concealment,” “blinding of the participants,” “blinding of the investigators,” “incomplete outcome data,” “selective outcome reporting” and “other bias”. Bias for each of the included study was rated as either “low risk,” “unclear risk,” or “high risk.” Green represents low bias, yellow represents ambiguity, and red represents high bias. When the evaluation satisfies complete low bias, it is grade A, indicating low bias; partly satisfies low bias, grade B, indicating moderate bias; and not satisfying at all, grade C, indicating high bias.

### Statistical analysis

We used RevMan 5.4 software to perform all statistical analysis. For measurement data, standardized mean difference was used as effect analysis statistic, and its 95% CI was provided. Heterogeneity among studies was explored using I^2^ statistic. Higgins et al. [26] developed a preliminary classification of I^2^ values with the purpose of aiding to interpret its agreement. Therefore, percentages of about 25% (I^2^ = 25), 50% (I^2^ = 50%), and 75% (I^2^ = 75%) would imply low, medium, and high heterogeneity, respectively. We conducted a random-effects meta-analysis in all cases. Reason of heterogeneity were judged by subgroup analysis, sensitivity analysis, or descriptive analysis. Stata12.0 was used for Begg’s and Egger’s test to quantify whether publication bias could have influenced the results. Linear relationship between global cognition and covariates was examined in meta-regression analysis. Meta-regression variables included: i) intervention duration, ii) year of publication, iii) assessment tools and country. A *p* value below 0.05 was considered statistically significant.

## RESULTS

### Study selection

**Fig. 1:**
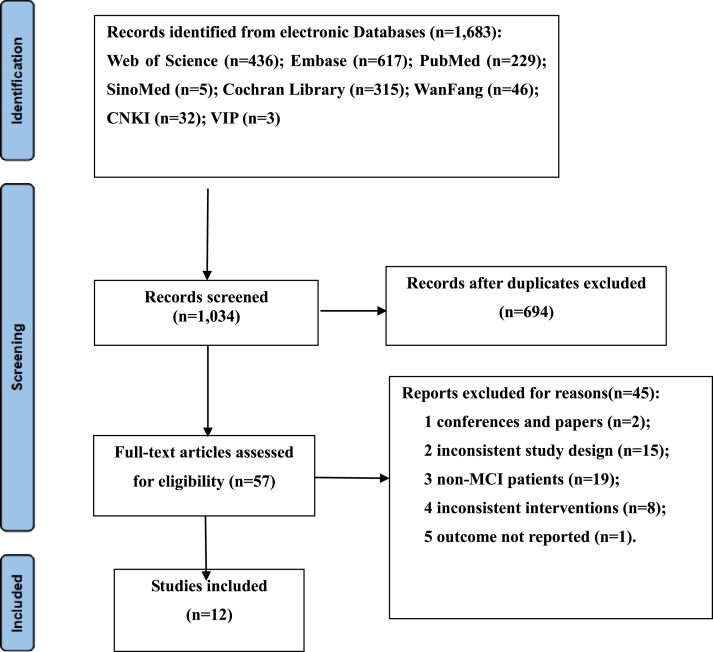
Flow diagram of the literature selection process.

The electronic search retrieved a total of 1,683 results. After removing duplicates, 1,034 records were screened by title and abstract. After the exclusion of irrelevant topics, the full texts of 57 articles were assessed for eligibility. A total of 44 studies were excluded because of conferences and papers (*n* = 2), inconsistent study design (*n* = 15), non-MCI patients (*n* = 19), inconsistent interventions (*n* = 8), the outcome and standard deviation was not displayed (*n* = 1). Finally, 12 [27–38] studies involving 1,124 elderly individuals (558 cases of intervention and 566 cases of placebo) were obtained in the final analysis. The results of the literature search and selection of included studies are presented in Fig. 1.

### Description of studies

Studies are detailed in Table 1. All studies investigated the effect of MCI due to DHA (alone or in combination with EPA) intervention among elderly subjects. Of these studies, 6 [27–31, 34] studies used Mini-Mental State Examination (MMSE), 4 [33, 35, 37, 38] studies used Chinese version of the Wechsler Adult Intelligence Scale-Revised (WAIS-RC), 1 [32] study used Basic Cognitive Aptitude Tests (BCATs), and 1 [36] study used Montreal Cognitive Assessment scale (MoCA) to assess subjects’ global cognitive function. Only four [28, 29, 31, 34] studies measured individual cognitive function. Four [27–29, 31] studies assessed patients’ depressive mood. Four [32, 35, 37, 38] studies measured changes in Aβ-related biomarkers and plasma inflammatory cytokines (Aβ_40_, Aβ_42_, AβPP, TNF-α, IL-6, IL-10) after intervention. We will describe quantitatively or qualitatively later. Two [28, 34] studies measured nutritional status of patients. Olfactory sensitivity was assessed by Rondanelli et al. [28]. Zhang et al. [33] assessed brain imaging. Nine [27, 29, 31–33, 35–38] studies assessed blood levels of DHA and/or EPA. The quality of the literature was assessed according to the Cochrane literature quality assessment tool, and all of the included literatures were of moderate quality (Table 2).

**Table 1:** Study characteristics

Study	Country	Diagnostic criteria	Sample Sizes (T/C)	Age mean (SD)	Intervention group (mg)	Duration of intervention (mo)	cognitive tools	Cognitive domains	Mood	blood biomarkers	Lever of DHA/EPA	adverse event
Chiu (2008) [27]	Taiwan, China	Petersen	17 /12	unclear	EPA1080mg+ DHA720mg	6	MMSE	Global	HDRS		Yes	soft stool or diarrhea, nausea, constipation
Rondanelli (2012) [28]	Italy	Petersen criteria	11 /14	85.3±5.3; 86.1±6.5	EPA286mg+ DHA720mg	3	MMSE; RAVLT; CDT; SVT	Global; Memory; CDT; SVT	GDS			No
Lee (2013) [29]	Malaysia	clinical diagnosis	17 /18	66.4±5.1; 63.5±3.0	EPA450mg+ DHA1300mg	12	MMSE; RAVLT; CDT	Global; Memory; CDT	GDS		Yes	swallowing difficulty and mild gastrointestinal discomfort
Mahmoud (2014) [30]	Iran	MMSE	40/40	unclear	EPA120mg+ DHA180mg	6	MMSE	Global				mild diarrhea
Phillips (2015) [31]	UK	Petersen criteria	37/39	71.1±8.6; 71.1±9.5	600mgEPA+ 625mgDHA	4	MMSES7; HVLT-R; CDT; SVT	Global; Memory; CDT; SVT	BASDEC		Yes	unclear
Zhang (2017) [33]	China	Petersen criteria	120/120	74.49±2.65; 74.57±3.31	DHA2000mg	12	WAIS-RC	Global			Yes	unclear
Li et al. (2021) [38]	China	DSM-5	60/60	71.55±6.62; 70.38±6.73	DHA 800 mg	6	WAIS-RC	Global		TNF-α, IL-6, IL-10	Yes	unclear
Zhang et al. (2018) [35]	China	Petersen criteria	120/120	73.71±2.24; 73.58±2.65	DHA2000mg	24	WAIS-RC	Global		Aβ_40_, Aβ_42_, AβPP, BACE1, APP mRNA	Yes	unclear
Baleztena (2018) [34]	Spain	Global Deterioration Scale; MMSE	34 /44	85.8±4.9; 87.8±6.5	EPA120mg+ DHA750mg	12	MMSE; CDT; SVT	Global£»CDT; SVT				difficulty to swallow and the excessive number of pills
Bo (2017) [32]	China	Petersen criteria	44/42	71.75±5.68; 70.45±6.82	EPA720mg+ DHA480mg	6	BCATs	Global		IL-6, IL-10, TNF-α,	Yes	unclear
Bai (2021) [37]	China	DSM-5	36/33	70.17±6.54; 68.30±6.38	DHA800mg	6	WAIS-RC	Global		Aβ_40_, AβPP, Aβ_42_, BACE1, APP mRNA	Yes	unclear
Wang (2021) [36]	China	Petersen criteria	30/30	69.20±4.89; 68.50±5.51	EPA720mg+ 480 mg DHA	2	MoCA	Global			Yes	difficulty to swallow

DSM-5, Diagnostic and Statistical Manual of Mental Disorders fifth edition; MMSE, Mini-Mental State Examination; GDS, Geriatric Depression Scale; RAVLT, Rey Auditory Verbal Learning Test; CDT, Clock drawing test; BASDEC, the Brief Assessment Schedule Depression Cards; WAIS-RC, Chinese version of the Wechsler Adult Intelligence Scale-Revised; SVT, The Semantic Verbal Fluency Test; BCATs, Basic Cognitive Aptitude Tests; MoCA, Montreal Cognitive Assessment scale; HVLT-R, the Hopkins Verbal Learning Test—Revised; blood plasma inflammatory cytokines: IL-6, Interleukin-6; IL-10, Interleukin-10; IL-1β, Interleukin-1β; TNF-α, tumor necrosis factor-α; Aβ-related biomarkers: Aβ_40_, Aβ_42_, AβPP.

**Table 2:**
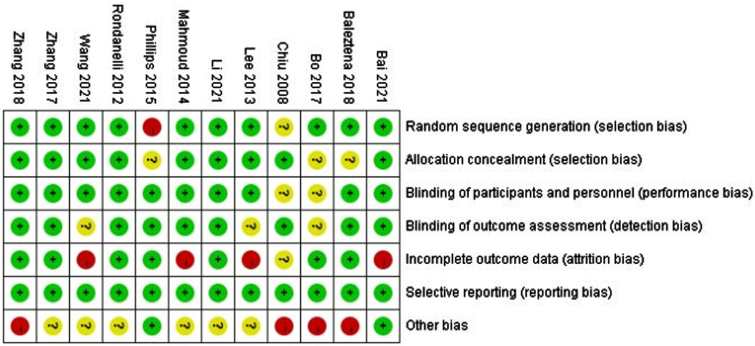
Risk of bias in the included studies

Green represents low bias, yellow represents ambiguity, and red represents high bias.

EPA and/or DHA were administered in 12 studies, and these were included in the meta-analysis. Four [33, 35, 37, 38] studies used only DHA as the n-3PUFAs intervention. Of the 12 studies, six [27, 29–31, 34, 36] studies reported no significant difference in the change in global cognitive function by n-3PUFAs supplementation compared with placebo. Improvement in the global cognition by EPA and/or DHA treatment was reported in other six [28, 32, 33, 35, 37, 38] studies. One [28] RCT reported an improvement in olfaction, and one [33] RCT showed significant differences in hippocampus of brain. Although five [27, 29, 30, 34, 36] studies reported the main complaints including the difficulty in swallowing the capsules and mild gastrointestinal discomfort (like soft stool, diarrhea, nausea, or constipation), all the studies’ compliance was high.

### The result of meta-analysis

#### Effects of n-3PUFAs supplements on global cognitive function

**Fig. 2:**
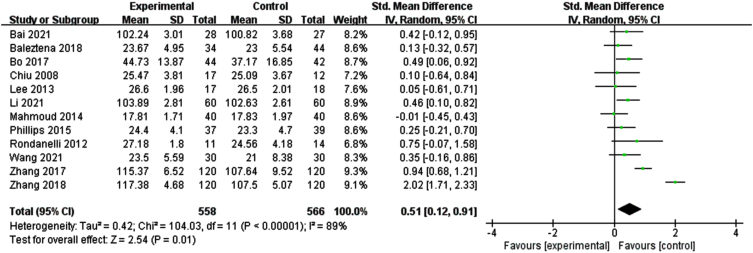
Forest plot for overall cognitive function. Test for heterogeneity I^2^ = 89%, *p* < 0.0001, the random effect model was used. The overall effect *p* = 0.01 <0.05, it shows that the intervention measures have a positive impact on the overall cognitive function of the elderly with MCI.

Figure 2 illustrates a forest plot for global cognitive function, showing a positive effect of n-3PUFAs treatment [SMD = 0.51, 95% CI (0.12, 0.91), *p* = 0.01]. However, a significant heterogeneity was found among the studies (I^2^ = 89%, *p* < 0.0001). Therefore, a random-effects model was used for meta-analysis.

#### Effects of n-3PUFAs supplements on memory

**Fig. 3A:**
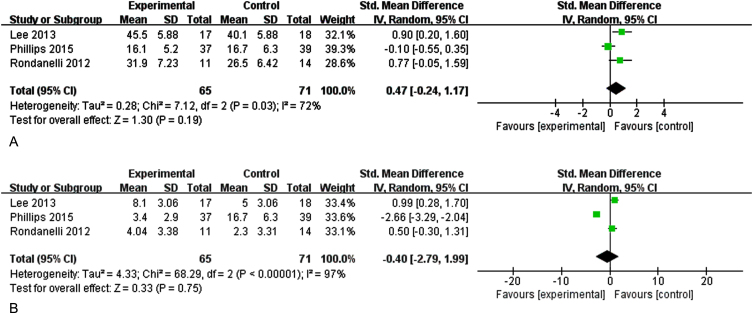
Forest plot for immediate memory. B. Forest plot for delayed memory. The effect of n-3CLPUFAs supplementation on memory (immediate and delayed memory) in older adults with MCI. A) Forest plot for immediate memory. Test for heterogeneity I^2^ = 72%, *p* = 0.03, the random effect model was used. The overall effect *p* = 0.19 >0.05, it shows that the intervention measures have no positive effect on immediate memory function of the elderly with MCI. B) Forest plot for delayed memory. Test for heterogeneity I^2^ = 97%, *p* < 0.001, the random effect model was used. The overall effect *p* = 0.75 >0.05, it shows that the intervention measures have no positive effect on delayed memory function of the elderly with MCI.

Three studies evaluated the effect of n-3PUFAs supplementation on memory (immediate and delayed memory) in elder adults with MCI (Fig. 3). Figure 3A shows the effect of n-3PUFAs on immediate memory. There was a high heterogeneity among the studies, and a random-effects model was selected for analysis (I^2^ = 72%, *p* = 0.03) [SMD = 0.47, 95% CI (–0.24, 1.17), *p* = 0.19]. Figure 3B shows the effect of n-3PUFAs on delayed memory (I^2^ = 97%, *p* < 0.0001) [SMD = –0.40, 95% CI(–2.97, 1.99), *p* = 0.75]. The results show that supplementation with n-3PUFAs did not improve memory in older adults with MCI.

#### Effects of n-3PUFAs supplements on other individual cognitive function

Phillips et al. [31], Rondanelli et al. [28], and Baleztena et al. [34] calculated the effect of supplementation with n-3PUFAs on language fluency in older adults with MCI. A positive trend for the semantic verbal fluency was found in the supplementation group, but the outcome did not have significant difference. Phillips et al. [31], Rondanelli et al. [28], Baleztena et al. [34], and Lee et al. [29] showed the supplementation with n-3PUFAs did not improve the executive functions of elderly people with MCI.

#### Effects of n-3PUFAs supplements on depression

**Fig. 4:**
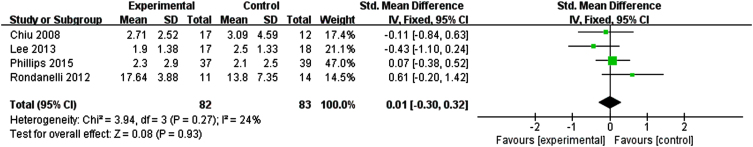
Forest plot for depression. Test for heterogeneity I^2^ = 24%, *p* = 0.27, the fix effect model was used. The overall effect *p* = 0.93 >0.05, it shows that the intervention measures have a positive impact on depression of the elderly with MCI.

Four studies evaluated the effect of n-3PUFAs supplementation on depressive mood. The results showed no significant effect to alleviate depression in older adults with MCI compared with the control group. Figure 4 shows the effect of n-3PUFAs on depression (I^2^ = 24%, *p* = 0.27) [SMD = 0.01, 95% CI(–0.35, 0.38), *p* = 0.93].

#### Effects of n-3PUFAs supplements on Aβ-related biomarkers and plasma inflammatory cytokines

Bai et al. [37] and Zhang et al. [35] explored the effects of DHA on blood amyloid-β (Aβ)-related biomarkers. There are only two articles on the Aβ-related biomarkers, and quantitative analysis may lead to misleading results. Therefore, we only give a brief description here. Zhang et al. [35] showed the Aβ_42_ level was lower in the intervention group than that in the control group, similar to the APP mRNA level. However, no significant differences in Aβ_40_ were observed. Bai et al. [37] indicated while DHA supplementation only led to a significant decline in Aβ_40_ level, no significant differences were observed in the Aβ_42_ and APP mRNA levels. Although they both measured β-secretase 1 (BACE1) and AβPP levels in blood, there was no statistical significance. Bo et al. [32] and Li et al. [38] assessed the effects of DHA and/or EPA intervention on the blood inflammatory cytokines in elderly subjects with MCI. Bo et al. [32] reported n-3PUFAs supplementation led to a significant decrease in 1L-6. Moreover, these two studies showed the intervention could reduce plasma TNF-α. Notably, these studies have also analyzed other indicators separately, but they cannot make inductive analysis.

### Effects on subgroups and sensitivity analysis

#### Subgroups analysis of global cognitive function

**Fig. 5:**
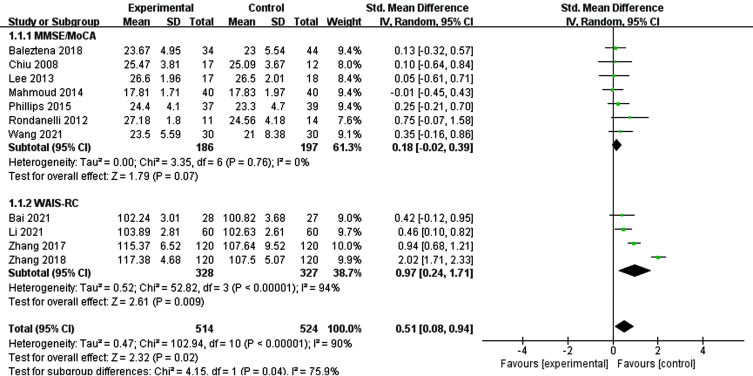
Subgroup analysis of overall cognitive function according to different assessment tools. For MMSE/MoCA, the test for heterogeneity I^2^ = 0%, *p* = 0.76.The overall effect *p* = 0.07 >0.05, the results are on the verge of being statistically significant. For WAIS-RC, the test for heterogeneity I^2^ = 90%, *P* < 0.001. The overall effect *p* = 0.02 <0.05, It shows that the intervention measures have a positive impact on global cognitive function of the elderly with MCI.

**Fig. 6:**
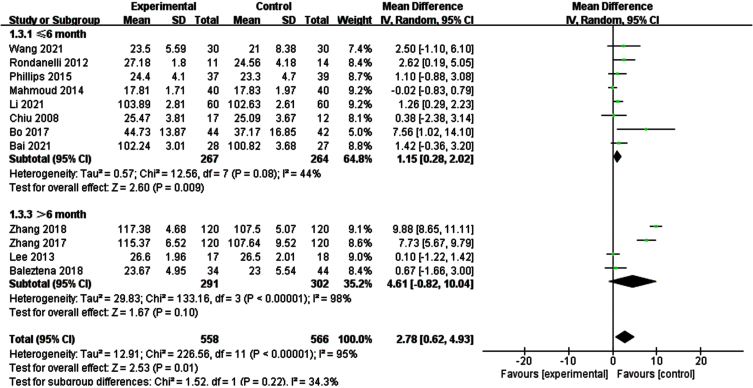
Subgroup analysis of overall cognitive function according to intervention duration. For ≤6 month, the test for heterogeneity I^2^ = 44%, *p* = 0.08.The overall effect *p* = 0.009 <0.05, the results shows supplementing n-3PUFAs ≤6 months has positive significance for the overall cognitive function of the elderly with MCI. For >6 month, the test for heterogeneity I^2^ = 98%, *p* < 0.0001. The overall effect *p* = 0.10 <0.05, It shows that the intervention measures have no positive impact on global cognitive function of the elderly with MCI.

Due to a significant heterogeneity was found on global cognitive function, we explored if different tools for assessing overall cognitive function altered the results by performing subgroup of studies, those MMSE/MoCA and those WAIS-RC (Fig. 5). The results of subgroup analysis were not significantly changed. Then, we conducted a subgroup analysis according to the duration of intervention (Fig. 6). The results showed that when the intervention time was less than 6 months, the heterogeneity between the intervention group and the control group was statistically significant (I^2^ = 44%, *p* = 0.08) [SMD = 1.15, 95% CI(0.28, 2.02), *p* = 0.009]. When the intervention time was more than 6 months, the heterogeneity was still large and had no statistical significance.

#### Sensitivity analysis of global cognitive function

**Fig. 7:**
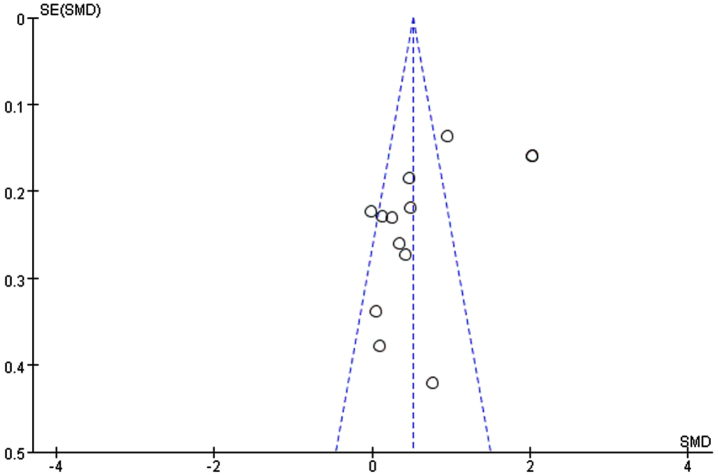
Funnel plot for global function. In the case of no bias, the figure is symmetrical inverted funnel; When there is publication bias, the funnel plot is asymmetric, and it is skewed.

**Fig. 8:**
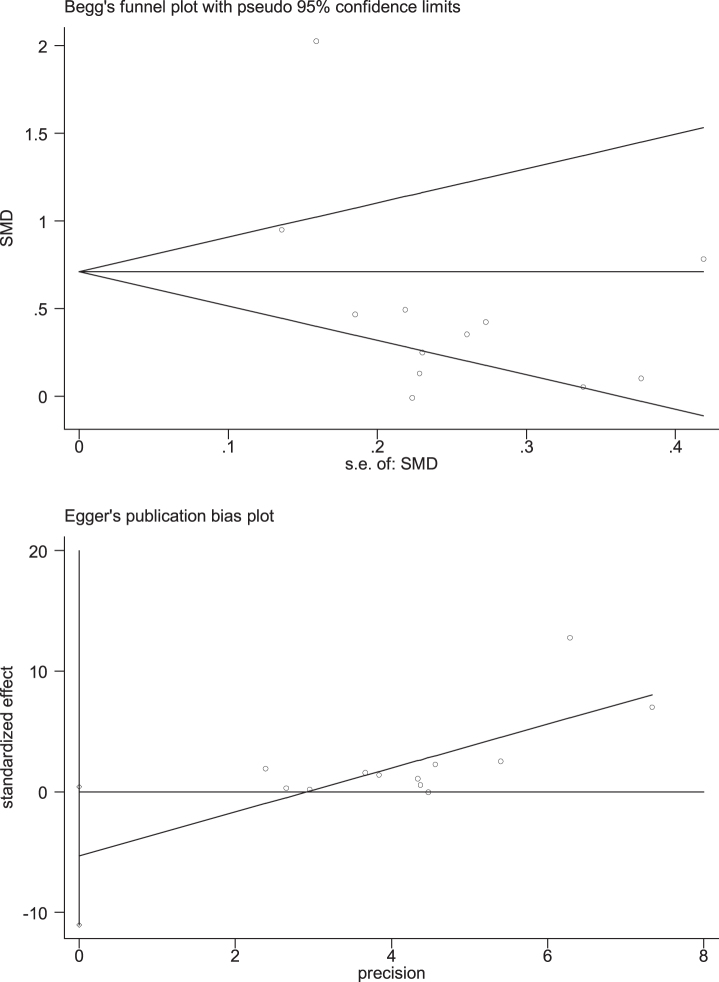
Egger’s and Begg’s test of global function. Begg’s test showed Pr > |z| = 0.631, Egger’s test showed *p* = 0.065, *p* > 0.05. The result showed there was no significant publication bias.

Through removing each study at a time in sensitivity analysis, we found that while the excluded studies changed the heterogeneity slightly, the results remain stable. That is, regardless of which article is excluded, our results still show that n-3PUFAs supplementation has a positive impact on the overall cognitive function of elderly with MCI. Funnel plot, a simple method to judge whether there is bias in meta-analysis, is mainly based on the degree of symmetry of the graph. There may be evidence of publication bias in the studies, since the funnel plot showed an asymmetry among selected studies (Fig. 7). Egger’s and Begg’s test were conducted to further quantify possible funnel plot asymmetry. Begg’s test showed Pr > |z| = 0.631, Egger’s test showed *p* = 0.065, *p* > 0.05. The result showed there was no significant publication bias (Fig. 8).

#### Sensitivity analysis of memory

**Fig. 9A:**
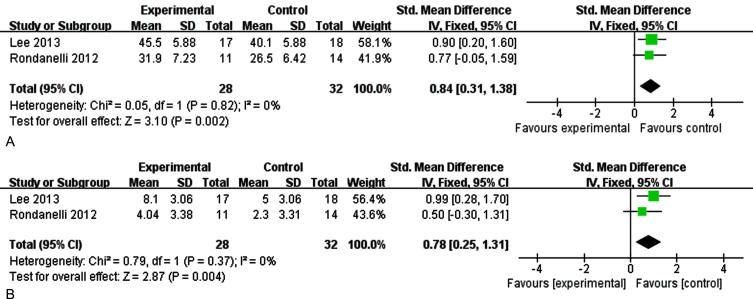
The sensitivity analysis for immediate memory. 9B. The sensitivity analysis for delayed memory. The sensitivity analysis for memory. A) The forest plot of sensitivity analysis for immediate memory, test for heterogeneity I^2^ = 0%, *p* = 0.82, the fixed effect model was used. The overall effect *p* = 0.002 <0.05, it shows that the intervention measures have no positive effect on immediate memory function of the elderly with MCI. B) The forest plot of sensitivity analysis for delayed memory, test for heterogeneity I^2^ = 0%, *p* = 0.37, the fixed effect model was used. The overall effect *p* = 0.004 <0.05, it shows that the intervention measures have positive effect on delay memory function of the elderly with MCI.

The outcome was memory in sensitivity analysis by removing each study at a time, when we excluded the study of Phillips et al. [31], there was no heterogeneity among the studies. Figure 9A showed the sensitivity analysis on immediate memory (I^2^ = 0%, *p* = 0.82) [SMD = 0.84, 95% CI(0.31, 1.38), *p* = 0.002]. Figure 9B reported the sensitivity analysis on delayed memory (I^2^ = 0%, *p* = 0.42) [SMD = 2.60, 95% CI(0.99, 4.21), *p* = 0.002]. The results show that supplementation of n-3PUFAs have a positive effect on memory function (immediate memory, delayed memory) of the elderly with MCI.

## DISCUSSION

The aim of this systematic review and meta-analysis was to evaluate the scientific evidence on the effects of n-3PUFAs supplementation on cognitive function, depression, Aβ-related biomarkers, and plasma inflammatory cytokines in MCI aged. There was a significant change in global cognitive function. After sensitivity analysis, the heterogeneity changed slightly for global cognitive function. However, considering the heterogeneity across studies, the results should be explained by great caution.

According to Fig. 5, we found 4 [33, 35, 37, 38] articles were the main reasons which caused heterogeneity. All these 4 articles used WAIS-RC to measure the overall cognitive function of the research subjects and the subjects were Chinese. We guess there may be regional differences. Subgroup analysis was conducted according to the intervention time, as shown in Fig. 6. We found that there was no statistical difference between the intervention group and the control group after the intervention time exceeded 6 months. Through reading the article, we found that it may be related to the MCI level and nutritional status of the subjects. This might be explained by the possibility of better nutrient synergies between the supplement and a good nutritional status. After sensitivity analysis by removing Phillips et al. [31], a significant statistical difference in memory function in those treated with n-3PUFAs was seen. We think that the data of individuals with cognitive impairment (not dementia) or with AD in early stage maybe combined due to the low recruitment in the latter population. As Canhada et al. [39] said, n-3 PUFAs may be beneficial in AD onset, but these data are not enough to support its therapeutic effect. Meta-regression analysis found that the year of public, region, intervention duration, and evaluation tools were not sources of bias (*p* > 0.05) (Table 3).

**Table 3:** The result of meta-regression about global cognition

ES	Coef.	Std. Err.	*t*	*p*> |t|	[95% Conf. Interval]	
Year	–0.0024026	0.0488874	–0.05	0.962	–0.118003	0.1131978
Duration	0.5440313	0.3302926	1.65	0.144	–0.2369865	1.325049
Tools	0.1823784	0.3334874	0.55	0.601	–0.606194	0.9709509
Country	0.4124811	0.4493805	0.92	0.389	–0.6501349	1.475097
Cons	3.691968	98.30118	0.04	0.971	–228.7534	236.1373

To date, the pathogenesis of dementia is unclear. A reduced level of DHA is associated with cognitive decline during aging. The roles and underlying mechanisms of DHA have been put forward. Neuroprotectin D1, a DHA derivative, may regulate brain cell survival and repair through neurotrophic, anti-apoptotic, and anti-inflammatory signaling pathways [40]. It is now clear that the levels of Aβ_40_, Aβ_42_, and other Aβ-related biomarkers in cerebrospinal fluid are useful for predicting the risk of MCI progressing into AD [41]. Our results also indicate that n-3PUFAs supplementation may have potential benefits for the elderly with MCI. Bai et al. [37] and Zhang et al. [35] showed that 6-month DHA supplementation could alleviate Aβ levels in elderly Chinese patients with MCI. However, there are only two articles on the Aβ-related biomarkers, and thus the mechanisms of n-3PUFAs supplementation on MCI need to be investigated in the future. Oulhaj et al. [42] showed DHA intervention combined with B vitamins or with folic acid have better effects on improving cognition and reducing dementia biomarkers than DHA alone [37]. Tokuda et al. [43] found that exercise with n-3PUFAs supplementation potentially improved attention and working memory in non-demented elderly Japanese individuals. These findings suggested the n-3PUFAs has synergistic effects for MCI patients when in combination with other measures. N-3PUFAs may also regulate MCI through inflammatory pathways [44]. A recent meta-analysis [45] reported that the levels of inflammatory markers in AD or MCI patients were different from that in normal people, supporting the notion that AD and MCI are accompanied by inflammatory responses in both the periphery and cerebrospinal fluid. In the studies we included, 2 [32, 38] studies reported n-3PUFAs can decrease certain plasma inflammatory cytokines in MCI individuals, but more research is needed in the future to clarify the relationship and mechanisms. Moreover, 4 [33, 35, 37, 38] studies reported supplementing DHA could benefits MCI, while DHA and/or EPA intervention did not alleviate depression symptom [27–29, 31]. Therefore, the results of DHA and/or intervention are affected by various factors, including supplementary measures, duration, and dosage, which needs further research.

Cerebral blood flow is essential to support neurons and other cells in brain, and disruption of cerebral blood flow may facilitate the development and progression of AD and other dementia [46]. Low cerebral perfusion may impair global cognitive function, memory, psychomotor speed, frontal lobe function and executive function [47]. Schwarz et al. [48] suggested that n-3PUFAs supplementation may potentially improve cerebral perfusion in patients who suffer from MCI, and thus have the potential to delay or even prevent further cognitive decline and the conversion to AD. Therefore, further intervention studies with larger sample size are necessary to investigate this promising therapeutic effect. Hippocampus plays a vital role in memory function [49]. DHA supplementation can significantly increase the volumes of hippocampus and global cerebrum and slow the progression of hippocampal atrophy [33]. Rondanelli et al. [28] assessed the nutritional status before and after the DHA intervention in patients. They found that there was only a significant improvement in MNA score, and the improvement of cognitive health can cause an amelioration of general well-being, which in turn increase nutritional status. Baleztena et al. [34] suggested an apparent improvement in memory loss if subjects were well nourished previously. Therefore, cognitive health and good nutritional status interact on each other. N-3PUFAs, as essential fatty acids, with only mild gastrointestinal discomfort [27, 29, 30, 34], should be investigated further to explore its benefits.

Nine [27, 29, 31–33, 35–38] studies showed the relationship between DHA and/or EPA levels after treatment compared with the control group, yet the measured results of DHA and/or EPA levels are controversial. Chiu et al. [27] reported n-3PUFAs supplementation increased DHA and total n-3PUFAs levels compared to placebo groups but did not altered the EPA level. Phillips et al. [31] showed DHA and EPA both increased after the n-3PUFAs supplementation for 1 month; however, there was no further increase 4 months later. Four [33, 35, 37, 38] articles show a significantly higher plasma DHA concentration in the DHA group than in the placebo group. Due to the variation in intervention dose and duration, it is plausible that a nonlinear relationship exists between n-3PUFAs brain levels and cognitive function. In addition, few of the included studies mentioned the intake of fish by the study subjects. Fish is rich in EPA and DHA, and fish consumption also appears to protect against dementia in the elderly [50]. This confounding factor should also be brought to the attention of investigators. Thus, the association between dietary n-3PUFAs deficiency and MCI is needed to be confirmed by more studies involving plasma n-3PUFAs levels in the future.

This systematic review and meta-analysis is comprised of several strengths. Firstly, we made a more comprehensive analysis of the intervention effect of n-3PUFAs supplementation on the elderly with MCI, including the possible impact on Aβ-related biomarkers and plasma inflammatory cytokines. There is no similar study before. Meanwhile, due to the lack of literature on Aβ-related biomarkers and plasma inflammatory cytokines, we need to further explore the internal mechanism of n-3PUFAs to improve the cognitive function of the elderly with MCI in the future. The relationship between the levels of EPA and DHA and the cognitive function of the elderly with MCI also needs further research. Of course, this document also has some limitations. First, there was a limited number of studies meeting our searching criteria, and this might affect the robustness of the results. Also, because the research subjects are more Chinese in the included literature published in recent years, due to different cultural backgrounds, it may also cause certain biases. Second, although the included literature is judged not to be the same study, but there is the same situation as the first author. It is not excluded that there is common research object. Finally, the quality of the included studies is mostly B grade, and the methodological quality needs to be further improved.

### Conclusion

This review indicates that treatment with n-3PUFAs results in a significant improvement in global cognitive function in old subjects with MCI. After sensitivity analysis, n-3PUFAs results in a small improvement in memory in MCI. N-3PUFAs may reduce Aβ-related biomarkers and plasma inflammatory cytokines in the elderly with MCI. However, due to the limited number of included literature, its mechanism needs to be further explored. Further studies are needed to assess the beneficial influence of n-3PUFAs levels on MCI. Large-scale randomized clinical trials are needed to further confirm our findings.

## Data Availability

The data supporting the findings of this study are available within the article. Further inquiries can be directed to the corresponding author.
